# A double jeopardy: transection of the aero-digestive tract after penetrating neck trauma

**DOI:** 10.11604/pamj.2018.30.185.14960

**Published:** 2018-06-28

**Authors:** Pietro Fransvea, Zamira Keyser

**Affiliations:** 1 Faculty of Medicine and Psychology, “Sapienza” University of Rome, St Andrea’s Hospital, Italy; 2 Trauma Unit, Tygerberg Hospital Cape Town, South Africa

**Keywords:** Penetreting trauma, areodigestive tract, surgery

## Image in medicine

A 32-year-old male patient presented to emergency department after sustained gunshot injury to the neck. Patient according to the advanced trauma life support (A.T.L.S.®) protocol appeared stable. The patient complained of dysphagia and odynophagia. Local examination revealed a gunshot entrance wound the left posterior neck triangle and no exit wound. A cervical bruising, subcutaneous emphysema was also noted. CT angiography of neck revealed a cut off of the right vertebral artery and extensive surgical emphysema which was suspicious for injury either the pharynx, oesophagus or trachea (A). A contrasted swallow revealed contrast extravasation consistent with hypopharyngeal/oesophageal injury (B). Given these findings, the patient was shifted to the operating room were a primary repair of the oesophagus and trachea was done with sternocleiod and omoiodeus muscle flap (A & B). Gunshot wound are still common worldwide especially in areas with high levels of criminal activities, gangsterism and lawlessness. In the past few years there has been a decline in the number of gunshot wounds seen at most major hospital but penetrating neck injuries are still commonly seen. Although many are minor injuries, they may deceptive in appearance and life threatening and the morbidity and mortality associated with blood loss and aerodigestive injuries remain significant.

**Figure 1:**
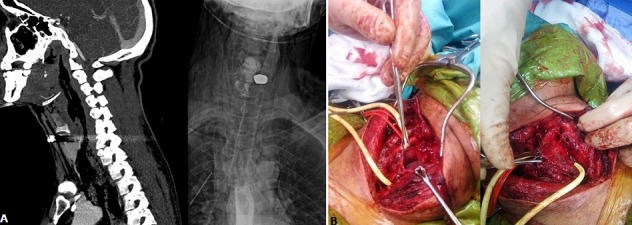
(A) imaging findings: extensive surgical emphysema contrast extravasation consistent with hypopharyngeal/oesophageal injury; (B) surgical findings: oesophagus and trachea transection

